# Effects of Loblolly Pine Biochar and Wood Vinegar on Poultry Litter Nutrients and Microbial Abundance

**DOI:** 10.3390/ani11082209

**Published:** 2021-07-26

**Authors:** Maryam K. Mohammadi-Aragh, C. Elizabeth Stokes, Jason T. Street, John E. Linhoss

**Affiliations:** 1 Department of Agricultural and Biological Engineering, Mississippi State University, Starkville, MS 39762, USA; john.linhoss@msstate.edu; 2 Department of Sustainable Bioproducts, Forest and Wildlife Research Center (FWRC), Mississippi State University, Starkville, MS 39759, USA; ces8@msstate.edu (C.E.S.); jts118@msstate.edu (J.T.S.)

**Keywords:** sustainable agriculture, wood waste, pyroligneous acid, waste management, nutrient retention, forest products, biomass

## Abstract

**Simple Summary:**

Developing a sustainable agroforestry system requires the utilization of byproducts. Poultry production and forestry are the two largest agricultural industries in Mississippi (USA) with loblolly pine being one of the most predominant timber species in the southeast. Poultry litter is a high-nutrient waste generated in poultry systems that is used as a fertilizer. However, poultry litter can release pollutants that reduce air and water quality and carries a heavy microbial load. Biochar and wood vinegar effects on nutrient retention and microbial activity vary due to the source material, production conditions, and application rate. This research evaluated how loblolly pine biochar and wood vinegar influence nutrients and microbial activity over time. The study found that wood vinegar reduced microbial growth in poultry litter-biochar blends. Fungi thrived in higher biochar proportions and bacteria preferred less biochar. Nitrogen and phosphorous were retained in 10% and 20% biochar blends. Furthermore, 10% and 20% biochar treatments were observed to be less odorous and compact, and the high pH of the biochar would be an effective conditioner for acidic soils. This study provides valuable information to stakeholders on how to utilize these materials to meet their objectives using regionally generated waste streams in the southeastern USA.

**Abstract:**

Biochar, wood vinegar, and poultry litter are waste streams that can be utilized as soil amendments and fertilizers. However, poultry litter releases several pollutants through nutrient leaching and carries heavy microbial loads, including potential human pathogens. Improving nutrient retention and reducing microbial load in poultry litter may help protect environmental and human health and improve its value as a soil amendment. The objectives of this study were to determine how blending varying proportions of loblolly pine (*Pinus taeda* L.) biochar, wood vinegar, and poultry litter affected nutrient profiles and microbial abundance over time. Biochar inclusion rates were 0%, 5%, 10%, and 20%, and wood vinegar was applied at 2% *w*/*w*. Samples were taken at Day 0, 57, and 112 to measure nitrogen, phosphorus, potassium, pH, total fungi, and total bacteria. Nutrient levels generally decreased with increasing biochar level; however, biochar inclusion rates of 10% and 20% retained nitrogen and phosphorus and exhibited improved physical properties. Overall, adding wood vinegar decreased nutrient concentrations and showed a biocidal effect for bacteria and fungi. Bacteria and fungi showed different relationships with biochar inclusion rates, with fungi preferring higher biochar inclusion rates and bacteria flourishing at lower biochar inclusion rates.

## 1. Introduction

Developing an environmentally sustainable agroforestry system requires the efficient utilization of waste streams from agriculture, forestry, and animal husbandry. Biochar (BC), wood vinegar (WV), and poultry litter (PL) are wastes generated in some forest products and poultry production facilities with the potential for numerous agricultural applications. Poultry production is the largest agricultural industry in Mississippi (USA), followed by forestry [1]. In 2019, approximately 756 million broilers were produced in Mississippi [1]. Currently, the most common method to dispose of PL is through direct land application as a fertilizer [2]. However, in some cases land application can lead to the leaching of nutrients into water systems, volatilization of greenhouse gases, and heavy metal deposits [3,4,5,6]. Furthermore, PL can release particulates, foul odors, and bio-aerosols that reduce air quality [7]. The numerous environmental and public health hazards generated from PL have necessitated the development of PL management and application strategies that reduce these hazards. Currently, byproducts from forest products industries are being evaluated as PL additives that may lessen the negative environmental and public health impacts. In this research, loblolly pine (*Pinus taeda* L.) BC and loblolly pine WV were evaluated to ascertain their effects on PL nutrient retention and microbial abundance over time.

BC is produced by subjecting biomass to high temperatures with little to no oxygen, known as pyrolysis [8]. Wood chips, shavings, bark, trimmings, and sawdust are byproducts of forestry and forest products industries that can by pyrolyzed to generate energy with BC left as a residue [9,10]. Around 97 million dry tons of wood residues are generated each year from the forestry and forest products industries, and loblolly pine is one of the most important commercial timber species in the southeastern U.S. [10,11,12]. Several studies have demonstrated that BC is an effective soil amendment and soil conditioner by reducing soil compaction and increasing soil stability, porosity, and water-holding capacity [13,14,15]. In addition, BC has been explored as an additive to reduce nitrogen loss through ammonia adsorption, aiding in nitrogen retention and reducing air pollution [10]. Although the nutrient and microbial impacts of BC addition to animal manures have been well-studied, the results are often varied and inconsistent depending on BC feedstock, inclusion rate, and application conditions. Therefore, providing information on how inclusion rates of loblolly pine BC impact factors such as nutrient retention and microbial activity will help stakeholders choose appropriate BCs, whether it be for PL storage, land application, or composting.

Several factors impact BC characteristics, including feedstock source, pyrolysis temperature, and pyrolysis type [16]. A meta-data analysis discovered that feedstock choice had the most influence on BC properties, with wood-derived BC having the greatest specific surface area and higher pore volume compared to crop residue, grasses, and manures/biosolids [16]. Specific surface area has been correlated with nutrient retention, and pore volume improves water availability and soil aeration [17,18]. Pyrolysis temperatures also has a significant impact on BC properties, with increasing temperatures resulting in higher alkalinity due to the detachment of acidic functional groups and the formation of negatively charged carboxyl and hydroxyl groups [19,20].

Studies have also shown that BC addition can induce significant changes to soil microbial communities [8,13]. Soil microorganisms are important for soil and plant health because they facilitate nitrogen fixation, nutrient cycling, defense mechanisms, and decomposition of soil organic matter [13]. Furthermore, studies have shown that BC stimulates microbial activity during composting, which promotes faster compost maturation [10,21,22]. However, BC inclusion rates of higher than 20% can negatively impact microbial activity [23,24].

Wood vinegar (WV), also known as pyroligneous acid, is a pyrolysis byproduct that can be isolated by distilling and clarifying vapors generated during pyrolysis [25]. It mostly consists of phenolic compounds, organic acids, and alcohols, which confer antimicrobial, antioxidant, and termiticidal properties, and the high concentration of acids gives WV a low pH (pH < 3) [26,27,28,29]. WV has also been used as an insect repellent, wood preservative, odor-remover, soil fertilizer, and animal-feed additive [30]. Although concentrated WV has biocidal properties, dilute doses stimulate microbial activity due to utilizing organic acids, aldehydes, and alcohols as carbon sources. [27,31,32,33]. WV has been shown to increase the maximum temperature during the thermophilic phase of composting, which is a result of increased microbial activity [22,34]. Research suggests that the effects of WV on physiochemical and microbial activity in soil and compost vary by feedstock; therefore, disseminating the effects of various WVs applied at different doses on microbial activity and soil nutrients is crucial to meet consumers’ objectives for downstream applications [35,36,37]. In addition, the low pH of WV would balance the alkalinity caused by BC addition and reduce nitrogen loss via ammonia volatilization.

BC’s and WV’s beneficial uses in soils and composts prompted this research to study the effects of loblolly pine (*Pinus taeda* L.) BC and WV on nutrient profiles and microbial abundance when blended with PL. BC and WV inclusion can provide improvements to soils, composts, and stored PL; however, determining the effects of BC and WV derived from various feedstocks and pyrolysis conditions are crucial for providing reliable and practical information to stakeholders. Therefore, the objectives of this research were to characterize how loblolly pine BC inclusion and WV addition influenced nutrient profiles and microbial abundance over time. This information will be valuable to forest products, agriculture, and poultry industry stakeholders for implementing PL management methods through adding value to used PL that will be used for composting or land application.

## 2. Materials and Methods

### 2.1. Experiment Setup and Sample Collection

A local forest products company donated loblolly pine BC (500 °C pyrolysis temperature). BC elemental analysis yielded 11.63% moisture content, 17.16% volatile matter, 3.00% ash, and 68.21% fixed carbon. The BC also contained 51.54% carbon, 4.78% hydrogen, 0.31% nitrogen, and 43.36% oxygen. WV (pH = 2.34) was produced by destructive distillation of loblolly pine from Living Web Farms in Hendersonville, NC, USA, and PL was collected from commercial broiler houses located on the campus of Mississippi State University. The litter was air-dried for 48 h, then PL and BC were combined to yield a total of 11 kg of dry material with eight treatments and five replicates (n = 40 treatment combinations) (Table 1). The total dry weight of 11 kg was chosen based on previous studies [38,39].

**Figure 1:**
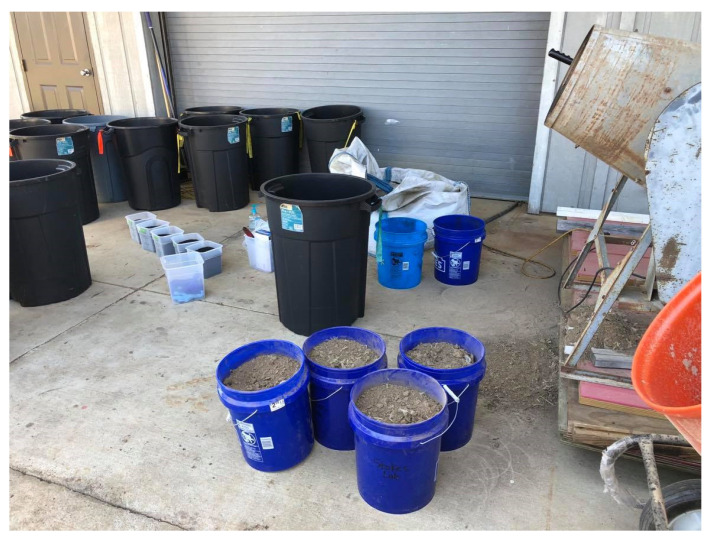
PL (blue buckets) were mixed with BC (clear containers) in a concrete mixer and transferred to perforated black 35-gallon containers.

Treatments were placed in 35-gallon heavy duty plastic bins (n = 40) purchased from a local hardware store. Five 3-cm holes were drilled into the bottom of the bins to allow excess water to drain, and a layer of water permeable landscape fabric (Sta-Green™, Lowe’s, Mooresville, NC, USA) was placed in the bottom of the bin to prevent materials from falling out. WV was applied at 2% *w*/*w*. PL and BC were blended in a concrete mixer for one minute and transferred to the bins (Figure 1).

A sample from each replicate was taken at Day 0 and measured for moisture content. The moisture content in each bin was adjusted to 50% using deionized water and WV if appropriate. No additional water was added during the experiment due to ample rainfall. The bins were transported to an outdoor site and randomly arranged on concrete blocks to facilitate drainage and were left uncovered. All bins were aerated at least once a week using a flathead shovel to promote aerobic conditions and to stabilize moisture content. The shovel was disinfected between treatments using 70% ethanol (EtOH). Samples were taken at Day 0, Day 57, and Day 112 to measure moisture content, pH, total fungi count, total bacteria count, and to conduct a nutrient analysis of nitrogen (N), inorganic phosphorus (P), potassium (K), and total carbon (C). After microbial enumeration tests were complete, samples were stored at −20 °C until ready for nutrient and pH analysis. These analyses were performed at the Mississippi State University Soil Testing Laboratory (Starkville, MS, USA).

### 2.2. Nutrient Analysis, pH, and Moisture Content

All samples were analyzed for nutrients, pH, and moisture content at the Mississippi State University Soil Testing Laboratory. Initial moisture content was recorded at the Mississippi State University’s Department of Sustainable Bioproducts (Starkville, MS, USA) to determine the amount of moisture to add to achieve a 50% moisture content for treatments. Both C and N were analyzed using an Elementar VarioMax C:N analyzer (Elementar Americas, Inc., Ronkonkoma, NY, USA). Inorganic P, K, and pH were determined using the standard soil analysis protocols used for the state of Mississippi.

### 2.3. Total Bacteria and Fungi Enumeration

Total fungi and bacteria enumeration were performed on each sample with three replicates. Serial dilutions were prepared by weighing 1 g of PL blend into 9 mL of sterile Difco ™ phosphate-buffered saline (PBS). Then, 1 mL of dilution mixture was transferred to respective growth media for fungi and bacteria. Day 0 fungi were cultured on potato dextrose agar with antibiotics (PDAA) plates (Becton, Dickinson and Company, Sparks, MD, USA) prepared as per the manufacturer’s instructions and incubated at 25 °C for one week. Day 57 and Day 112 fungal enumerations were performed using total yeast and mold (YM) petrifilms (3M^TM^ Petrifilm^TM^, St. Paul, MN, USA). YM petrifilms were incubated at 25 °C and were enumerated after 72 h as per the manufacturer’s instructions. Day 112 YM petrifilms were repeated due to no growth after 72 h. All bacteria enumeration samples were cultured on Aerobic Count Plate (ACP) petrifilms (3M^TM^ Petrifilm^TM^, St. Paul, MN, USA) as per the manufacturer’s instructions. The ACP petrifilms were incubated at 30 °C for 24 h and then enumerated. The total number of colonies in a sample was calculated by multiplying the number of colonies on the film by its dilution factor.

### 2.4. Observational Physical Characteristics of Poultry Litter, Biochar, and Wood Vinegar Mixtures

Notable physical characteristics were recorded for PL blends over the course of the experiment, including texture, odor, insect activity, and moisture. Texture was noted during sample collection on Day 57 and 112 and was categorized by the degree of compaction. Insect activity was recorded during aeration of PL treatments and included the observable presence and abundance of insect larvae and pupae within the PL treatments. Observable differences in moisture or water accumulation among treatments was noted during weekly aeration. Although the records were not quantifiable, the observations provided insights into how biochar altered the physical attributes of the PL mixtures.

### 2.5. Statistical Analysis

The split-plot design model was used to determine significant treatment interactions with a 5% level of significance. BC and WV were the main plot factors, and time was the subplot factor. PROC GLM was run on a single response variable (e.g., nitrogen concentration), and main plot factors that exhibited a significant interaction with time (*p* < 0.05) were run through PROC MIXED METHOD = TYPE3 to account for treatment factors for BC, WV, and time. Response variables that showed significant interactions with BC × time or WV × time were run through the same procedure as independent fixed effects. LSMEANS was performed on significant interactions at a 5% level of significance.

## 3. Results

### 3.1. pH, C:N, and Nutrient Analysis

#### 3.1.1. pH

**Figure 2:**
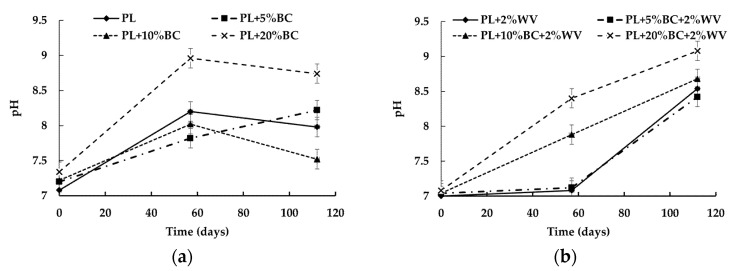
pH values for treatments without WV (**a**) and with WV (**b**) over time.

A significant relationship was found (*p* = 0.0419) between pH, BC level, WV, and time. pH increased significantly over time for all treatments except PL + 5% BC and PL + 5% BC + 2% WV. WV treatments showed a higher end point pH compared to treatments without WV (Figure 2).

In general, increasing BC level and WV inclusion resulted in higher pH values, with PL + 20% BC + 2% WV having the most alkaline pH at 9.08. PL and PL + 5% BC were not significantly different from each other at Day 112, and WV inclusion did not result in a significant difference in pH for treatments containing 5% and 20% BC. At Day 112, PL + 20% BC + 2% WV was the only WV treatment to have a significantly different pH for WV treatments at Day 112, as PL + 2% WV, PL + 5% BC + 2% WV and PL + 10% BC + 2% WV clustered at an approximate pH of 8.4.

#### 3.1.2. C:N Ratio

**Figure 3:**
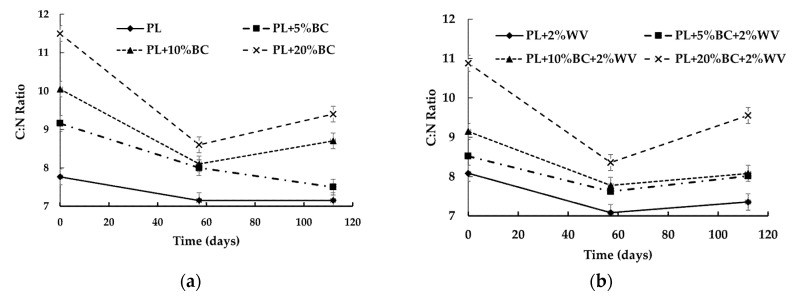
C:N ratios for treatments without WV (**a**) and with WV (**b**) over time.

There was a significant interaction between the combination of BC, WV, and time for C:N ratio (*p* = 0.0454). The C:N ratios significantly decreased from Day 0 to Day 112 for all treatments, and PL + 20% BC/20% BC + 2% WV had significantly higher C:N ratios at the end of the study compared to other BC treatments, but were not significantly different from each other (Figure 3).

#### 3.1.3. Nitrogen

**Figure 4:**
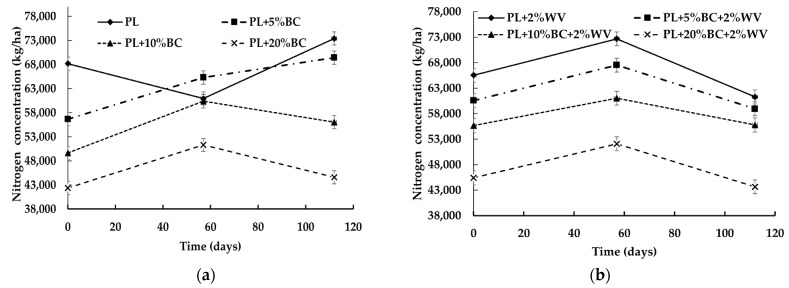
Nitrogen concentration (kg/ha) for poultry litter blends without WV (**a**) and with WV (**b**) over time.

The combination of BC level, WV, and time influenced total nitrogen (N) concentration (kg/ha) at a statistically significant level (*p* < 0.0001). Overall, all treatments except the control increased in total N from Day 0 to Day 57 (Figure 4).

After Day 57, PL and PL + 5% BC significantly increased in N. Treatments containing 10% and 20% BC (with and without WV) lost a significant amount of N between Day 57 and Day 112; however, none of these treatments experienced a significant N loss at the end of the study. This indicates that 10% and 20% BC inclusions rates may have helped retain N. Furthermore, at Day 112, PL + 10% BC showed significantly higher N levels compared to Day 0. PL, PL + 5% BC, and PL + 10% BC had significantly higher N concentrations at Day 112 compared to the beginning of the study, with the control having the highest N concentration. Overall, N concentration significantly decreased as BC level increased. For WV treatments, PL + 2% WV and PL + 5% BC + 2% WV began with significantly higher N concentrations than PL + 10% BC + 2% WV and PL + 20% BC + 2% WV; however, PL + 2% WV lost a significant amount of N by the end of the study. BC aided in retaining N in treatments containing WV, because PL + 2% WV was the only treatment to lose a significant amount of N over time.

#### 3.1.4. Phosphorus and Potassium

**Figure 5:**
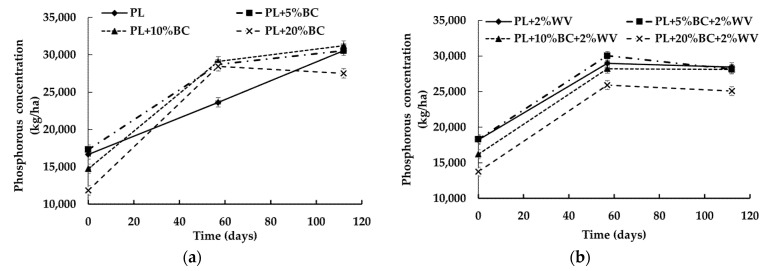
Phosphorous concentration (kg/ha) for poultry litter blends without WV (**a**) and with WV (**b**) over time.

The combination of BC level, WV, and time influenced phosphorous (P) concentration (kg/ha) at a statistically significant level (*p* = 0.0003). All treatments showed a significant increase from Day 0 to Day 57 (Figure 5).

BC treatments without WV and PL increased in P from Day 57 to Day 112 except for PL + 20% BC. Overall, adding WV resulted in significantly lower P concentrations at Day 112 when compared to P concentrations of treatments without WV. WV treatments had significantly lower final P concentrations at the end of the study compared to treatments without WV. Therefore, the addition of WV decreased the concentration of inorganic P in PL blends. PL + 5% BC + 2% WV was the only treatment that significantly decreased in P between the Day 57 and Day 112.

No significant interactions were found for potassium (K) concentrations (kg/ha) and BC levels or WV (*p* = 0.3301). However, K concentrations decreased over time (Table 2). Manures typically contain plenty of K for plant health, so K concentrations are generally not an issue when animal manures are used as fertilizer [40].

### 3.2. Microbial Enumeration

**Figure 6:**
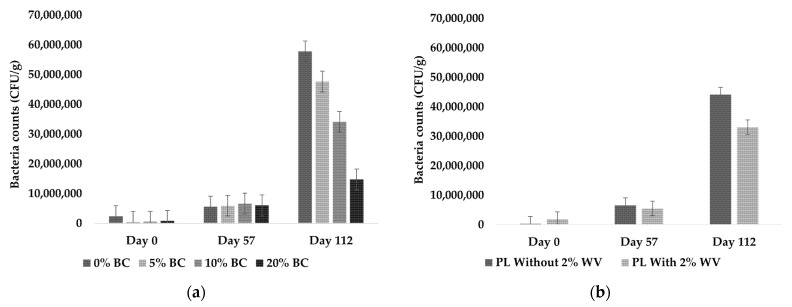
Bacteria counts for all BC inclusion rates, including treatments with and without WV (**a**), and WV level (**b**) as independent fixed effects.

Bacteria counts were not significantly affected by the combination of WV and BC over time; therefore, BC and WV had independent fixed effects on bacteria counts over time. BC level and WV inclusion significantly affected bacteria counts at *p* < 0.0001 and *p* = 0.0322, respectively. Bacteria counts did not significantly increase between Day 0 and Day 57, but all treatments significantly increased between Day 57 and Day 112 (Figure 6).

At Day 112, bacteria counts were significantly decreased with increasing BC levels (Figure 6a). At Day 112, treatments containing WV had significantly lower bacteria counts than treatments without WV (Figure 6b). This indicates that loblolly pine WV had an antibacterial effect when applied at 2%.

**Figure 7:**
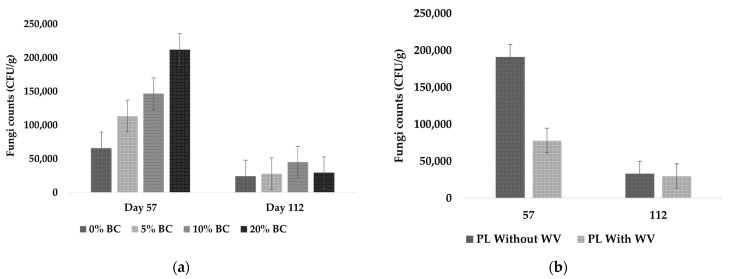
Fungi counts for all BC inclusion rates, including treatments with and without WV (**a**), and WV level (**b**) as independent fixed effects.

Fungi counts were significantly affected by WV over time (*p* = 0.0020) and the BC × time effects were near the significance level (*p* = 0.0596). Because of the inherent variation in conducting outdoor experiments, BC × time was included as a significant interaction. BC and WV had independent fixed effects on fungal counts over time. Fungal counts were significantly higher at Day 57 for all treatments and counts significantly decreased afterwards (Figure 7a). Wood vinegar showed a biocidal effect on fungi counts, with treatments containing WV having significantly lower fungal counts (Figure 7b).

### 3.3. Observational Physical Characteristics of Poultry Litter, Biochar, and Wood Vinegar Mixtures

**Figure 8:**
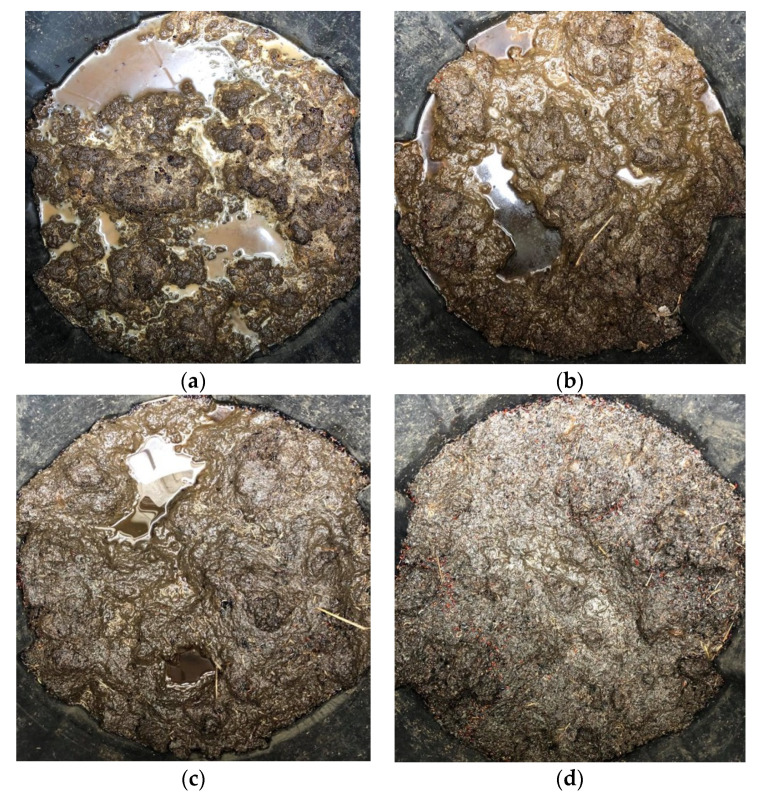
Water accumulation in PL blends for treatments containing 0% BC (**a**), 5% BC (**b**), 10% BC (**c**), and 20% BC (**d**). Less standing water was observed in treatments containing more BC, with 20% BC showing no observable standing water.

Insect larvae and pupae were found in all blends in the first two weeks of the experiment. After the first two weeks, less insect activity was observed in treatments containing 10% and 20% BC. Increasing the BC level appeared to reduce insect activity, which is a favorable quality for storing materials containing animal manure [41]. The WV did not appear to influence insect activity. Some treatments were not draining efficiently after a prolonged period of heavy rainfall, which resulted in the accumulation of standing water. The amount of standing water decreased with increasing proportions of BC (Figure 8).

**Figure 9:**
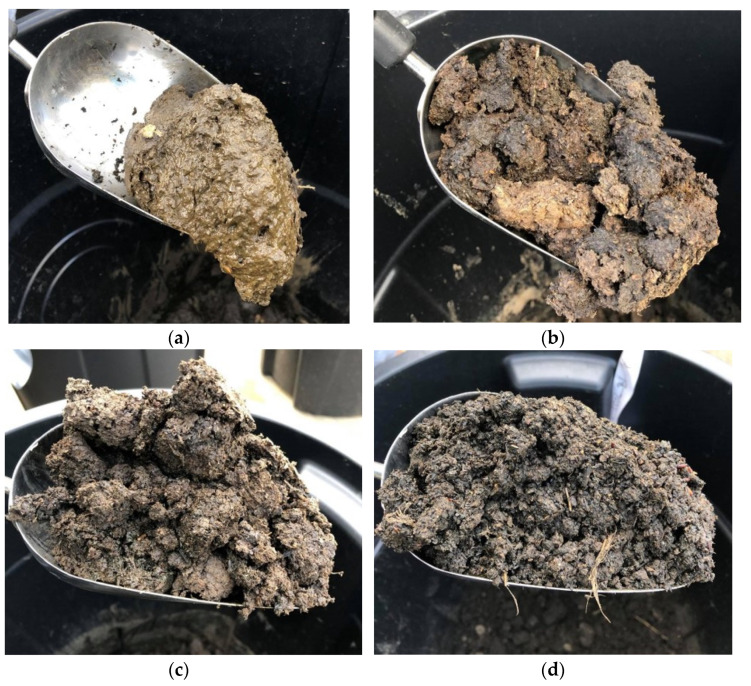
Final collection of PL treatments containing 0% BC (**a**), 5% BC (**b**), 10% BC (**c**), and 20% BC (**d**). PL blends were less compacted with increasing levels of BC.

PL, PL + 2% WV, PL + 5% BC, PL + 5% BC + 2% WV, and some PL + 10% BC/PL + 10% BC + 2% WV treatment containers were not draining well due to water not permeating through the fabric. PL and PL + 2% WV had slurry or sludge-like consistencies, and it is likely that fine PL particles clogged the fabric. BC may have reduced compaction due to its rigid, porous structure, which reduces compaction. Moreover, BC increases water holding capacity (WHC) [15,42]. Yu et al. (2013) found that 9% BC addition doubled the WHC of loamy sand soils and Linhoss et al. (2019) reported that 20% BC inclusion rate in PL significantly increased WHC when compared to 0% to 10% inclusion rates. In addition, they estimated that a 20% BC inclusion rate in a 15.2 m × 152.4 m commercial broiler house could increase WHC of litter by 49,210 L. These results further demonstrate the beneficial physical properties of including BC in PL and BC’s effectiveness as an absorbent in PL matrixes. During the final sample collection, the texture and odor of the various treatments were noted. Treatments containing 20% BC were fine textured and nearly odorless. Treatments blended with 5% and 10% BC exhibited a compacted, clumpy, and sticky texture with some foul odor. Treatments containing no BC were sludge-like and emanated intensely foul odors. Therefore, as the BC level increased, the texture became notably less compacted and less odorous (Figure 9).

## 4. Discussion

The measured increase in pH due to BC is in accordance with a study that reported a significant increase in pH with 10% BC addition to pig manure compost [34]. In addition, the authors found that 2% WV inclusion did not significantly impact compost pH; however, the present study found that WV significantly increased pH values for PL + 2% WV and PL + 10% BC + 2% WV treatments at the end of the study compared to the non-WV treatments with the same BC level (*p* = 0.0057 and *p* < 0.001, respectively). Increases in pH could also be a result of ammonia accumulation, while decreases in pH could be caused by nitrification, ammonia volatilization, or the production of organic acids [22]. The decrease in pH from Day 57 to Day 112 for BC treatments without WV could be a result of the significant increase in bacterial activity, which released acidic metabolites. WV inhibited microbial growth; therefore, less acid compounds would have been produced, and may explain why WV treatments were more alkaline. In the non-WV treatments, it is possible that bacteria would have recovered once mixtures dried out and increased cellular activity. Furthermore, the C:N ratio may provide indications of N cycling that is reflected in the pH and N concentrations. For example, as pH increased from Day 0 to Day 57 for all treatments, the C:N ratio decreased. This may indicate nitrification was occurring, resulting in the accumulation of nitrate, which contributed to an increase in pH over time and simultaneously lowered the C:N ratio. This is also reflected in the general increase in N between Day 0 and 57. After Day 57, the elevated pH could have resulted in N volatilization, which raised the proportion of C in the mixtures. PL and PL + 5% BC, and PL + 2% WV and PL + 5% BC + 2% WV tended to cluster together for pH values, C:N ratios, and N concentration at Day 112. This further illustrates the relationship between pH, C:N ratio, and N cycling, and how the levels of loblolly pine BC and WV impacted these factors.

A significant decrease in C:N ratio over time was expected because microorganisms break down carbon sources [43]. Additional carbon sources would be needed to compost PL–BC blends, which requires a C:N range of 20:1 to 40:1 [44,45]. Although BC is mostly comprised of carbon, its stability makes it resistant to microbial decomposition [46]. PL and PL + 2% WV were not significantly different from each other at Day 112, indicating that WV addition did not have a substantial impact on C:N ratio over time.

The high pH of PL + 20% BC may have favored ammonia volatilization, which could result in lower final N concentrations. Because PL began with a low C:N due to being 100% PL, it is possible that more N volatilized from these treatments, resulting in an initial decrease in N [41]. Less N may have volatilized in BC treatments between Day 0 and Day 57 due to adsorption to the BC surface and to the higher C:N ratio favoring N retention. The adsorption of N may have also reduced volatilization despite high pH values for BC treatments at Day 57. Other factors that may have impacted the nutrient results are the clogged fabric at the bottom of the containers or that BC addition diluted the amount of N in the matrix.

The clogging of the fabric at the bottom of the containers in PL/PL + 2% WV and PL + 5% BC/PL + 5% BC + 2% WV treatments suggests that nutrient leaching was impeded. Because PL + 10% and 20% BC (with and without WV) treatments had improved drainage, it is possible that nutrients could have leached through the fabric, which would result in lower nutrient concentrations for reviewers (with and without WV) treatments. Without measuring the leachates, the amount of N, P, and K that were lost through leaching cannot be determined. However, despite the unfavorable conditions for N retention, such as the alkaline pH and potential nutrient leaching, N concentrations were not significantly different at the end of the study from Day 0 for PL + 10% BC + 2% WV, PL + 20% BC, and PL + 20% BC + 2% WV. Furthermore, PL + 10% BC increased significantly in N from Day 0 to Day 112. Therefore, higher BC inclusion rates may have aided in N retention, although they were lower than other treatments overall. The decrease in N from Day 0 to Day 57 for the control could be attributed to a low C:N ratio that favored ammonia volatilization [41]. In BC treatments, N could have been retained due to adsorption to the BC surface and to the higher C:N ratio of BC treatments. Although the pH was higher for BC treatments, it is possible that adsorption aided in preventing N loss. PL + 2% WV was the only treatment that lost a significant amount of N over the course of the study, suggesting that adding 2% loblolly pine WV to PL without BC addition may not promote N retention.

One possible reason for the decrease in phosphorous in PL + 20% BC is leaching, as P is not lost through volatilization [47]. The P concentrations followed a similar trend as N in treatments with WV. The P concentrations in WV treatments were significantly lower than treatments without WV. The increase in P indicates that the organic P in the litter was being transformed into inorganic forms, which is the only form that is plant available [48]. Recycling P has become a critical topic of interest in sustainable agriculture because P is mainly derived from non-renewable phosphate rock [49]. The demand for inorganic P fertilizer has increased due to the rapidly growing world population, and phosphate rock is being mined faster than it is being replaced [50,51]. Another source of P is animal manure, and some research proposes pyrolyzing animal manures to provide inorganic P in agricultural soils [52,53,54]. However, this would involve expensive and time-consuming logistics such as transportation, locating pyrolysis facilities, and coordinating with end users. Furthermore, the multivalent metal cations in charred material reduces P solubility compared to uncharred material, which supports that adding BC to manures may be more beneficial than converting manures to BC [53,55]. A meta-analysis conducted by Glaser and Lehr (2019) on BC effects on P availability in agricultural soils reported that BC application to acidic (pH < 6.5) and neutral soils (pH 6.5 to 7.5) showed positive effects on P availability. In the meta-analysis, 48 out of 54 BCs were alkaline, indicating that BC has a liming effect in acidic soils, thereby improving nutrient use efficiency [56,57,58]. In the present study, the increased alkalinity of higher BC levels is in accordance with this information. Therefore, 10% and 20% BC inclusion rates would be beneficial for conditioning acidic soils.

BC production from wood feedstocks is currently a process utilized by some forest products’ manufacturing processes. This available BC would benefit poultry producers that apply BC to the poultry house to reduce odor and litter moisture and adds value to the spent litter due to increased nutrient retention. In this system, waste streams are being recycled to improve crop production rather than creating a new waste product (e.g., pyrolysis of animal manures).

The significant increase in bacteria counts by the end of the study was expected; however, it was projected that bacterial abundance at Day 57 would be significantly higher than Day 0. There are several potential causes for low bacterial abundance at Day 57, including water logging that led to anaerobic conditions, cool temperatures, competition with fungi, and the high alkalinity in PL + 20% BC and PL + 20% BC + 2% WV. High alkalinity (>9.0) causes denaturation of enzymes and other proteins involved in metabolic pathways, which would inhibit bacterial growth [59]. Mixtures containing no BC showed significantly higher bacteria counts than other treatments at Day 112. BC appeared to have an inhibitory effect on bacteria counts, because every increasing level of BC resulted in significantly lower bacteria colony forming units (CFU). However, it is also possible that adding BC diluted total number of bacteria in 11 kg of starting material, resulting in lower bacteria counts with increasing BC inclusions.

PL blends with WV showed significantly lower bacteria counts. This indicates that loblolly pine WV used at a 2% application rate may inhibit bacterial growth. Studies have shown that the effects of WV on microbial activity is dose dependent, with low doses stimulating microbial activity and higher doses inhibiting microbial growth [28,32,33]. A study found that adding WV at 0.26% *w*/*v* (pH = 2.04) did not significantly impact microbial activity [21]. Another study reported that pig manure composts treated with 2.0% WV, 10% BC, and 10% zeolite reached thermophilic temperatures faster than controls [34]. Because microbial activity is the primary driver of compost temperatures, the rapid temperature rise in the study is most likely attributed to microbial activity [41]. However, the WV feedstock was not reported, and some comparisons cannot be made due to the inherent variation in WV feedstock chemical composition [36,37].

Fungi enumeration media was changed from PDAA to YM petrifilms due to a prolonged incubation time, laborious process, and consuming a significant portion of laboratory space. Because a variation analysis was not performed on YM petrifilms and PDAA, the results are incomparable and Day 0 fungi counts were removed.

Unlike bacteria, fungal abundance significantly increased as the level of BC increased (Figure 6a). Fungal counts were highest at Day 57 for all treatments, which could have presented nutrient competition for bacteria and resulted in low bacteria counts for that collection period. Fungi are more tolerant of pH extremes, which may have resulted in an advantage for colonizing higher BC levels [59]. Although loblolly pine BC appeared to have stimulated fungal growth, WV showed an inhibitory effect on fungal abundance. As the study progressed the fungal abundance waned, and bacterial growth significantly increased. By the end of the study, fungal abundance had decreased significantly. The drastic increase in bacteria likely provided nutrient competition that resulted in lower fungal counts. Several studies have reported similar findings, with soil microbiomes shifting towards bacterial dominance over time [60,61,62].

## 5. Conclusions

1. Loblolly pine WV was effective for significantly reducing overall microbial load at a 2% inclusion rate.
2. Although PL blends had significantly lower concentrations of N and P as BC level increased, BC treatments either increased in N and inorganic P or retained concentrations that were not significantly different from the beginning of the study.
3. Loblolly pine BC addition to PL increased fungal abundance.
4. Loblolly pine BC can alter the physical characteristics of PL.

## Figures and Tables

**Table 1:** Amounts of poultry litter and biochar used for treatments.

Treatment	Biochar (kg)	Poultry Litter (kg)	WV (kg)
PL	0	11	-
PL + 5% BC	0.55	10.45	-
PL + 10% BC	1.1	9.9	-
PL + 20% BC	2.2	8.8	-
PL + 2% WV	0	11	0.18
PL + 5% BC + 2% WV	0.55	10.45	0.18
PL + 10% BC + 2% WV	1.1	9.9	0.18
PL + 20% BC + 2% WV	2.2	8.8	0.18

**Table 2:** Potassium concentrations (kg/ha) over time for poultry litter blends.

	Day 0	Day 57	Day 112
PL	52,590	30,995	26,896
PL + 5% BC	52,723	38,920	29,621
PL + 10% BC	53,994	26,951	27,471
PL + 20% BC	43,752	23,273	21,348
PL + 2% WV	56,472	29,954	22,953
PL + 5% BC + 2% WV	59,508	26,525	22,880
PL + 10% BC + 2% WV	51,618	24,022	21,974
PL + 20% BC + 2% WV	43,003	18,048	17,096

## Data Availability

Data is available upon request from the corresponding author and pending agreement by co-authors.
